# Baseline subcutaneous adipose tissue volume as a potential determinant of lymphedema manifestation

**DOI:** 10.3389/fphys.2026.1993536

**Published:** 2026-10-29

**Authors:** Hiroo Suami, Atsushi Fujita

**Affiliations:** 1 Australian Lymphoedema Education, Research and Treatment (ALERT) Centre, Faculty of Medicine, Health and Human Sciences, Macquarie University, Sydney, NSW, Australia; 2 Veterinary Medical Center, Graduate School of Agricultural and Life Sciences, University of Tokyo, Tokyo, Japan

**Keywords:** animal model, fixed-skin animal, indocyanine green lymphography, loose-skin animal, lymphedema, subcutaneous adipose tissue

## Abstract

Lymphedema is a pathological condition characterized by swelling of the affected body region and debilitating discomfort due to functional impairment of the lymphatic system. Despite its clinical significance, the pathophysiology underlying its onset and the factors regulating its severity remain poorly understood. Its markedly higher prevalence in women has yet to be explained. Lymphedema is traditionally described as a consequence of imbalance between interstitial fluid production and lymphatic drainage capacity. However, no established method currently exists to quantify either of these volumes. In this article, we aimed to address several unresolved aspects of lymphedema pathophysiology, including the inability to develop a reliable animal model, the vulnerability of women, the different presentations of the affected limb between lean and overweight patients, and the onset of primary lymphedema during puberty. We used imaging data as supportive evidence and proposed an anatomy-based hypothesis in which the baseline volume of subcutaneous adipose tissue may determine lymphedema development and its manifestation as a clinically measurable parameter.

## 1 Introduction

Chronic edema occurs when interstitial fluid production exceeds clearance by the lymphatic system, resulting in fluid accumulation within tissues (Mortimer and Levick, 2004). Lymphedema, which is a subtype of chronic edema, is attributed to impaired lymphatic transport due to structural or functional disorders of the lymphatic system (Mortimer and Rockson, 2014). Although lymphedema is most commonly observed in the extremities, it also occurs in the head and neck following cervical lymph node dissection, and in the breast after breast‐conserving surgery (Deng et al., 2012; Brunelle et al., 2024).

Lymphedema is categorized clinically into two types: secondary lymphedema associated with surgical intervention, trauma, or other specific history causing lymphatic insufficiency, and primary lymphedema, which lacks any antecedent events. This classification is based solely on medical history and does not consider underlying pathophysiology. Early symptoms include swelling of the local region due to lymphatic stasis and a sensation of heaviness or discomfort. In lower limb lymphedema, patients initially notice difficulty wearing their previously fitting shoes due to ankle swelling. Prolonged lymphedema leads to skin thickening, subcutaneous tissue fibrosis and adipose deposition (Mortimer and Rockson, 2014; Executive Committee of the International Society of Lymphology, 2020).

Several studies have examined risk factors for the development of secondary lymphedema and identified a number of factors, such as surgical interventions, radiation treatment and obesity (DiSipio et al., 2013; Decorte et al., 2025). However, the fundamental determinants of its onset remain unclear from a pathophysiological aspect. Classical classification of primary lymphedema divides cases into three types: congenital, praecox, and tarda, based on age of onset (Kinmonth, 1965). Congenital cases develop lymphedema from birth to two years old and are caused by developmental disorders of the lymphatic system. Praecox cases are the most common type, and their onset is from adolescence to 35 years old. Though maldevelopment of lymphatic vessels and hormonal effects are assumed, the pathological mechanisms of the delayed onset are unspecified. Epidemiological studies revealed that lymphedema exhibits a pronounced sex difference, with women predominating across both primary and secondary types (Smeltzer et al., 1985; DiSipio et al., 2013). While breast‐cancer–related and gynecologic‐cancer–related lymphedema contribute to this trend, primary lymphedema also occurs more frequently in women, and the basis for this sex difference remains unexplained.

Animal models are essential for elucidating disease mechanisms. Despite relentless attempts, researchers have been unable to develop reliable animal models that can reproduce persistent, human-like limb swelling accompanied by chronic adipose and fibrotic tissue remodeling. The rodent tail model is widely used with circumferential skin excision (Slavin et al., 1999; Tabibiazar et al., 2006). However, its feasibility to mimic human limb lymphedema is questionable.

In this paper, we propose an anatomy-based hypothesis about these unresolved questions regarding lymphedema using imaging data in animals and humans as supportive evidence. A part of interstitial fluid is composed of a metabolic product of tissues, and lymphedema primarily affects the skin and subcutaneous tissue above the deep fascia. We hypothesize that baseline subcutaneous adipose tissue volume correlates with the amount of interstitial fluid production and may influence the development of lymphedema. As such, subcutaneous adipose tissue has the potential to be used as a measurable parameter in clinical settings.

## 2 Relevant findings

### 2.1 Anatomic differences between humans and animals

Scientists have attempted to develop a lymphedema model in animals over decades. Earlier studies frequently used canines, whereas recent advances in molecular biology have shifted focus toward rodent models (Frueh et al., 2016). Canine models often mimicked human oncologic procedures, including lymph node dissection and radiation therapy. However, the canine models failed to demonstrate persistent limb swelling associated with adipose hypertrophy. Although there was no volume increase, lymphangiography in postoperative dogs demonstrated significant lymphatic structural changes like human lymphedema, including dermal backflow and lymphatic regeneration (Danese et al., 1962; Suami et al., 2016). Rodent hind-limb lymphedema models also demonstrated transient swelling lasting approximately one month (Bucan et al., 2024). The rodent tail model is currently a promising model. However, although this model is useful for physiological and pharmacological studies, its anatomical relevance to human limb lymphedema is doubtful (Frueh et al., 2016).

A key anatomical distinction between humans and animals is the structure of their skin. Rodents, dogs, and cats are categorized as “loose-skin animals,” in which the dermis is weakly connected to underlying tissues and the skin is movable. In contrast, humans and pigs are called “fixed-skin animals.” Loose-skin animals have very thin layers of subcutaneous adipose tissue, conceivably indicating lower interstitial fluid production than fixed-skin animals. In previous canine studies, acute edema occurred after lymphatic injury (**Figure 1**) (Suami et al., 2016). The swelling gradually improved after a few months, but lymphangiography revealed marked lymphatic alterations resembling human lymphedema (**Figure 2**). An MRI image of a normal limb demonstrates that the subcutaneous adipose volume is far less than that in humans (**Figure 3**). These findings suggest that even when lymphatic structural changes and functional impairment occur, the lower interstitial fluid load in loose-skin animals may be adequately managed, resulting in a higher threshold for lymphedema development. The distinction of loose versus fixed skin may be considered an important anatomical contributor to interspecies differences and help explain why chronic secondary lymphedema cannot be reliably reproduced in these species. Conversely, if we focus solely on lymphatic morphological changes rather than limb volume, these canine models may be considered successful in representing human lymphedema.

**Figure 1:**
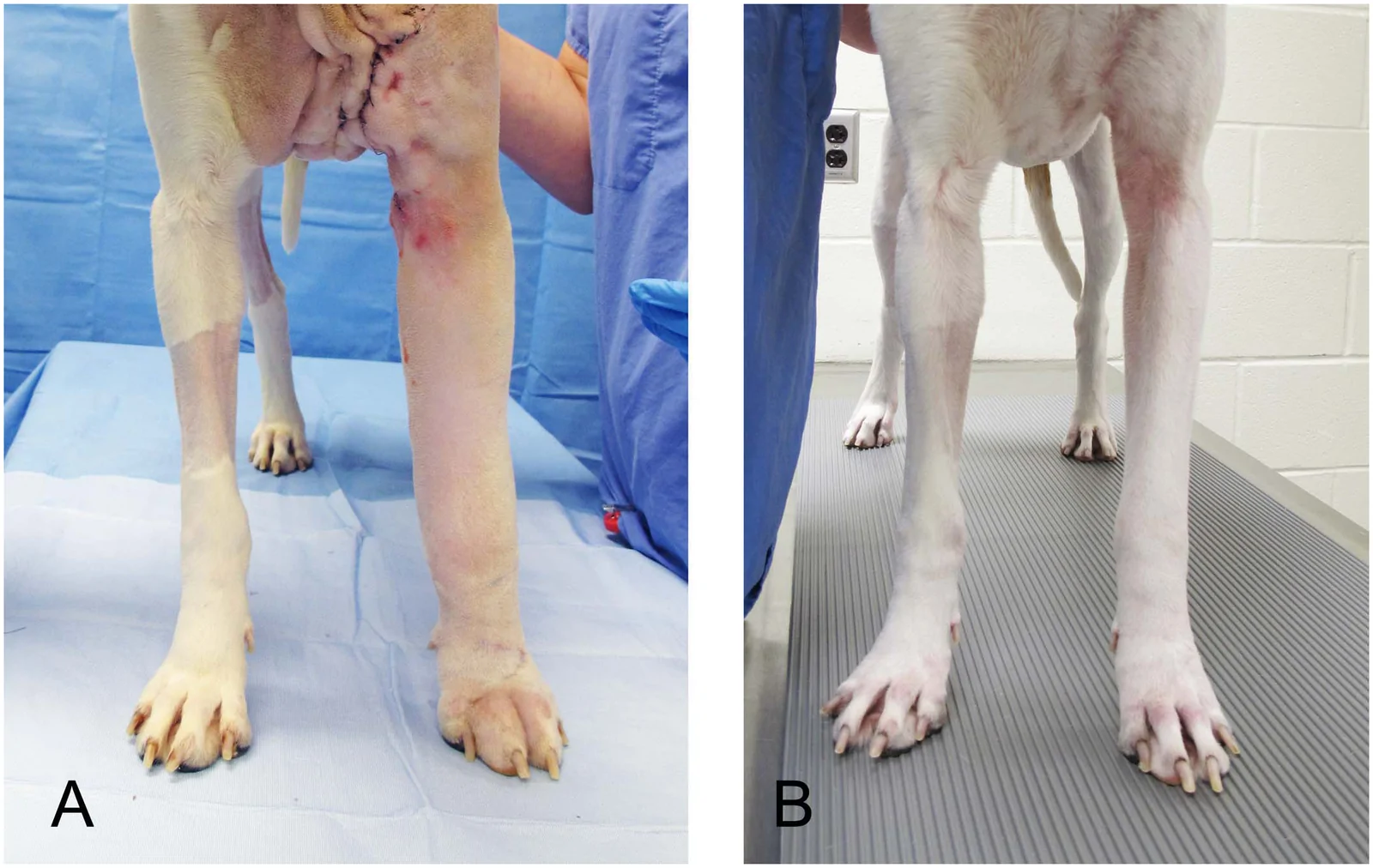
The left canine forelimb was swollen one month after lymph node dissection and irradiation **(A)**. The swollen forelimb recovered to its original size three months post-surgery **(B)**. Photos reproduced from Suami et al. (2019) with permission from Hiroo Suami.

**Figure 2:**
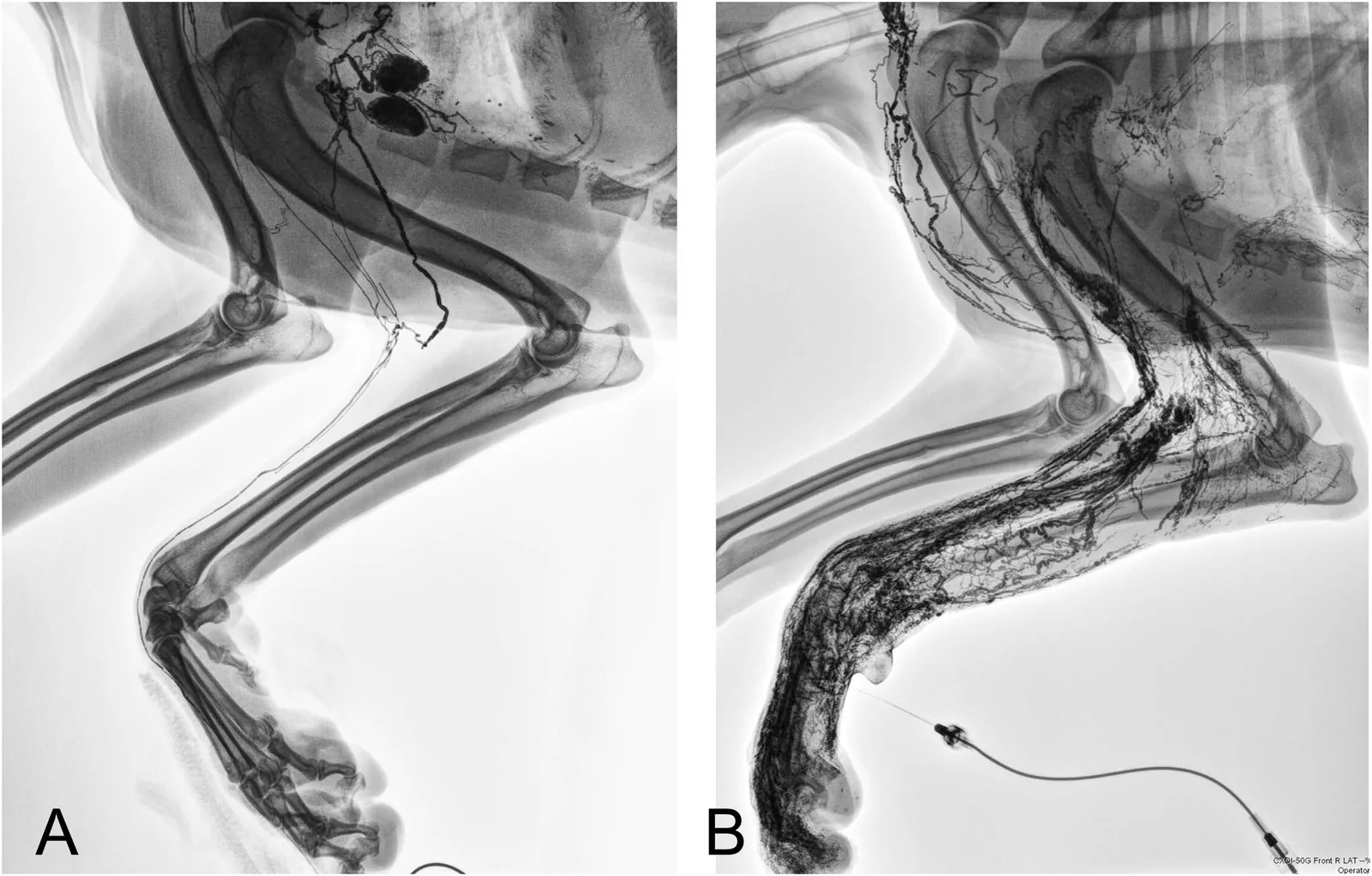
Radiographic lymphangiography images in the left forelimb before surgery **(A)** and nine months post-surgery and irradiation **(B)**. Linear collecting lymph vessels and axillary lymph nodes were demonstrated before surgery **(A)**. Extensive backflow was present in the entire left forelimb **(B)**, similar to human lymphedema. Photos reproduced from Suami et al. (2019) with permission from Hiroo Suami.

**Figure 3:**
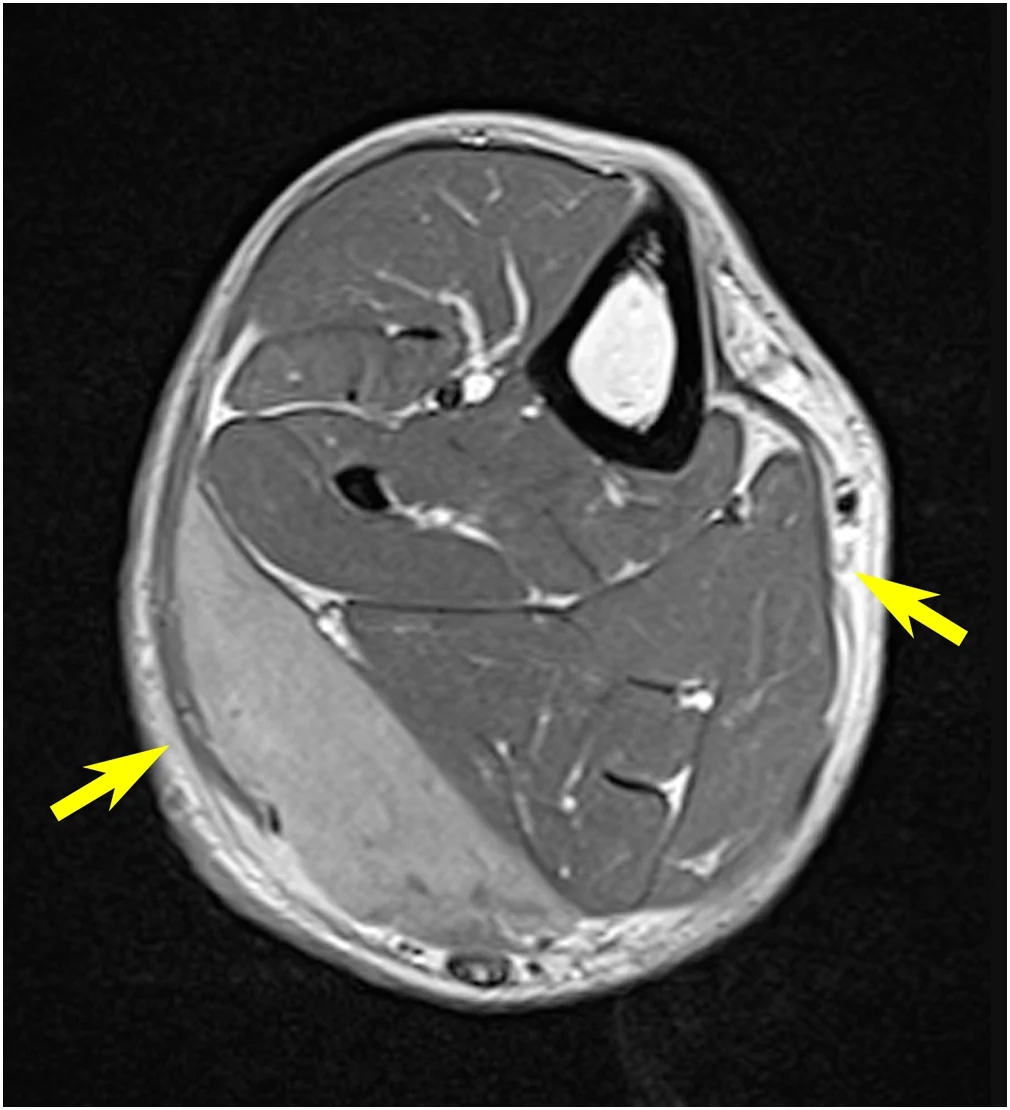
T1 MRI image of a canine hind limb. The subcutaneous adipose tissue layer is very thin (arrows).

### 2.2 Lymphedema manifestation between lean and overweight individuals

Overweight or obesity is a well-recognized risk factor for lymphedema, and weight control is recommended as part of management (DiSipio et al., 2013; Decorte et al., 2025). Obesity impairs multiple aspects of lymphatic function: it increases lymphatic vessel permeability, reduces the pumping rate of collecting vessels, and is associated with chronic inflammation and fibrosis (Nitti et al., 2016). However, lymphedema also occurs in lean individuals, though its phenotypic characteristics have not been well described.

Circumferential tape measurement or volume measurement is a standard assessment tool for limb lymphedema because of its simplicity (Karges et al., 2003). Additionally, indocyanine green (ICG) lymphography has been used for diagnostic imaging assessment of lymphedema at the Australian Lymphoedema Education, Research and Treatment clinic (Suami et al., 2019, 2022). We observed a discrepancy in lean patients between the stage of their lymphedema and the amount of swelling: despite advanced lymphedema on ICG imaging (MD Anderson Cancer Center Stage 3 and 4), there was minimal limb enlargement (**Figures 4**, **5**). In overweight patients, the limb enlargement was much more pronounced (**Figures 6**, **7**). This discrepancy suggests that the baseline volume of subcutaneous adipose tissue in patients with lower body weight may mitigate the amount of overt, clinically apparent swelling in lymphedema cases, even when there is substantial structural alteration.

**Figure 4:**
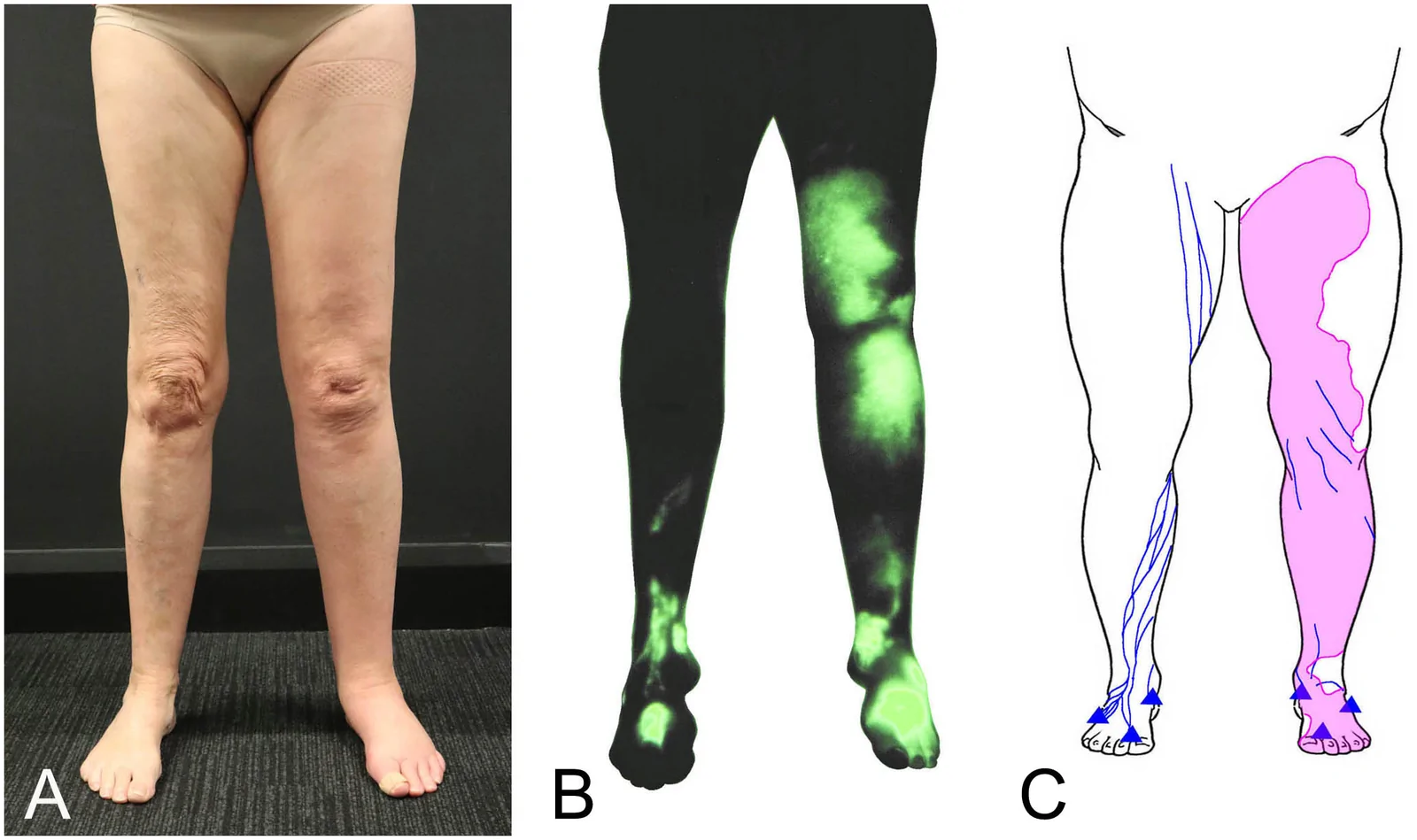
ICG lymphography images of a lean female patient (BMI 21) with unilateral secondary lymphedema in the left lower limb (MDACC Stage 3). From left: patient photo **(A)**, ICG montage image **(B)** and schematic diagram **(C)** (collecting lymph vessel: blue, dermal backflow: pink).

**Figure 5:**
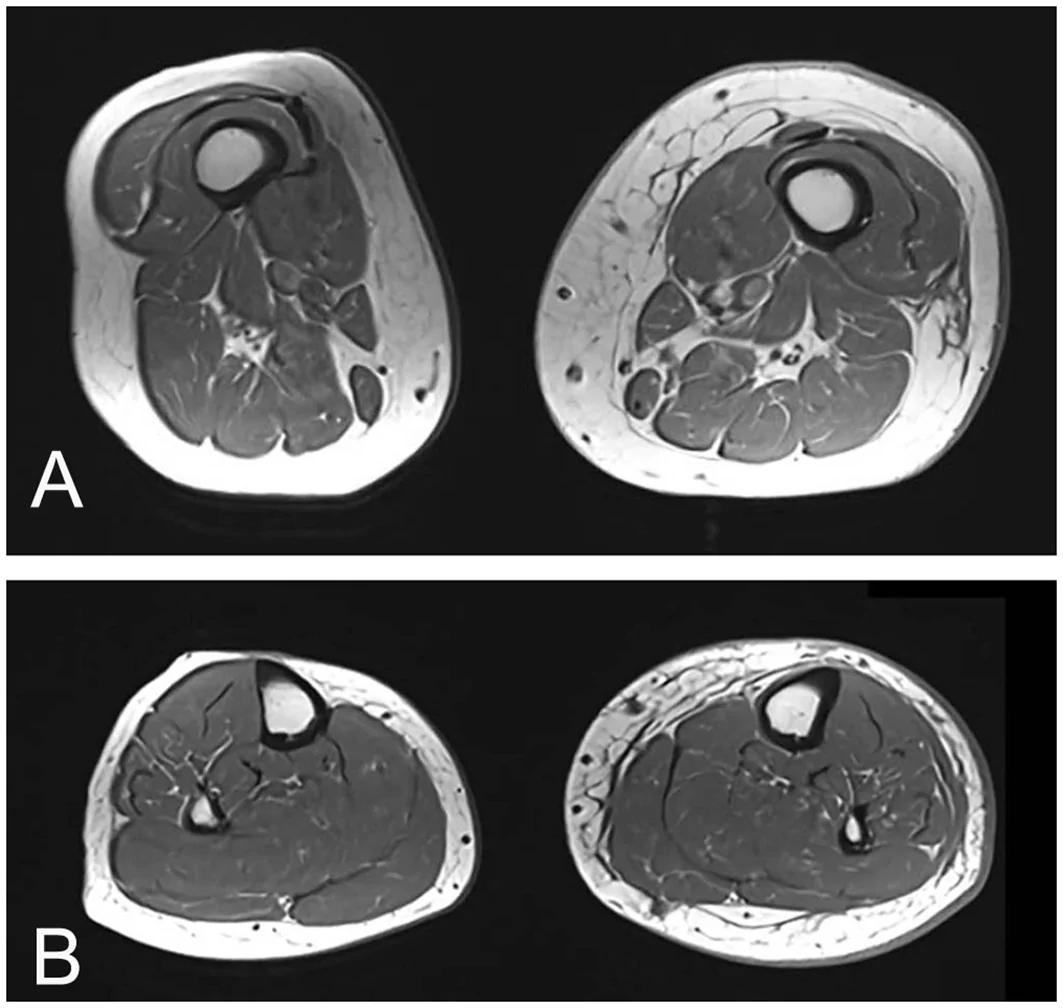
T1 MRI images of the lymphedema patient in **Figure 4**, 30 cm above the knee **(A)** and 30 cm below the knee **(B)**. The left lower limb volume is 9,347 mL and the right lower limb is 8,255 mL, a volume difference of 16%.

**Figure 6:**
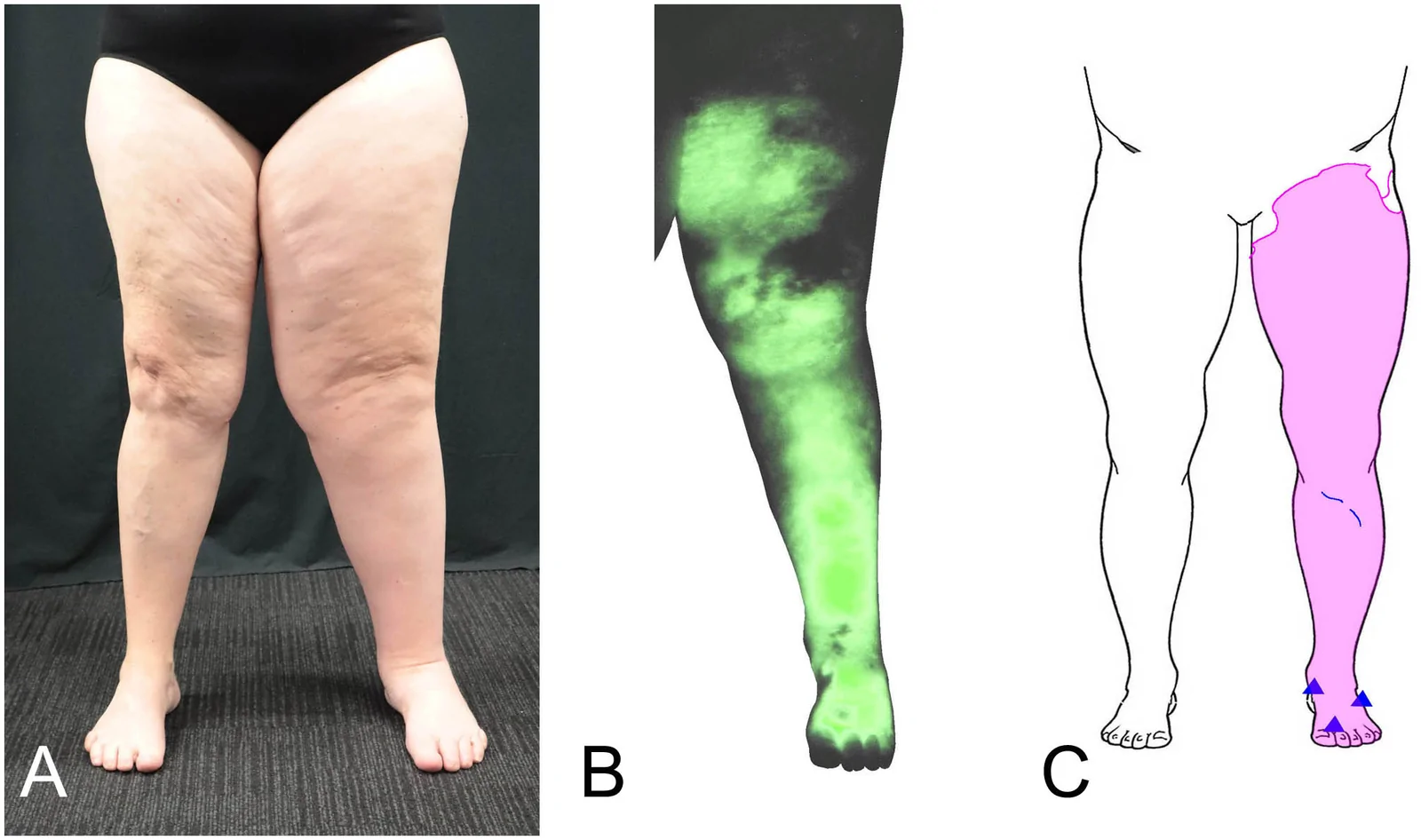
ICG lymphography images of an overweight female patient (BMI 35) with unilateral secondary lymphedema in the left lower limb (MDACC Stage 4). From left: patient photo **(A)**, ICG montage image **(B)** and schematic diagram **(C)** (collecting lymph vessel: blue, dermal backflow: pink).

**Figure 7:**
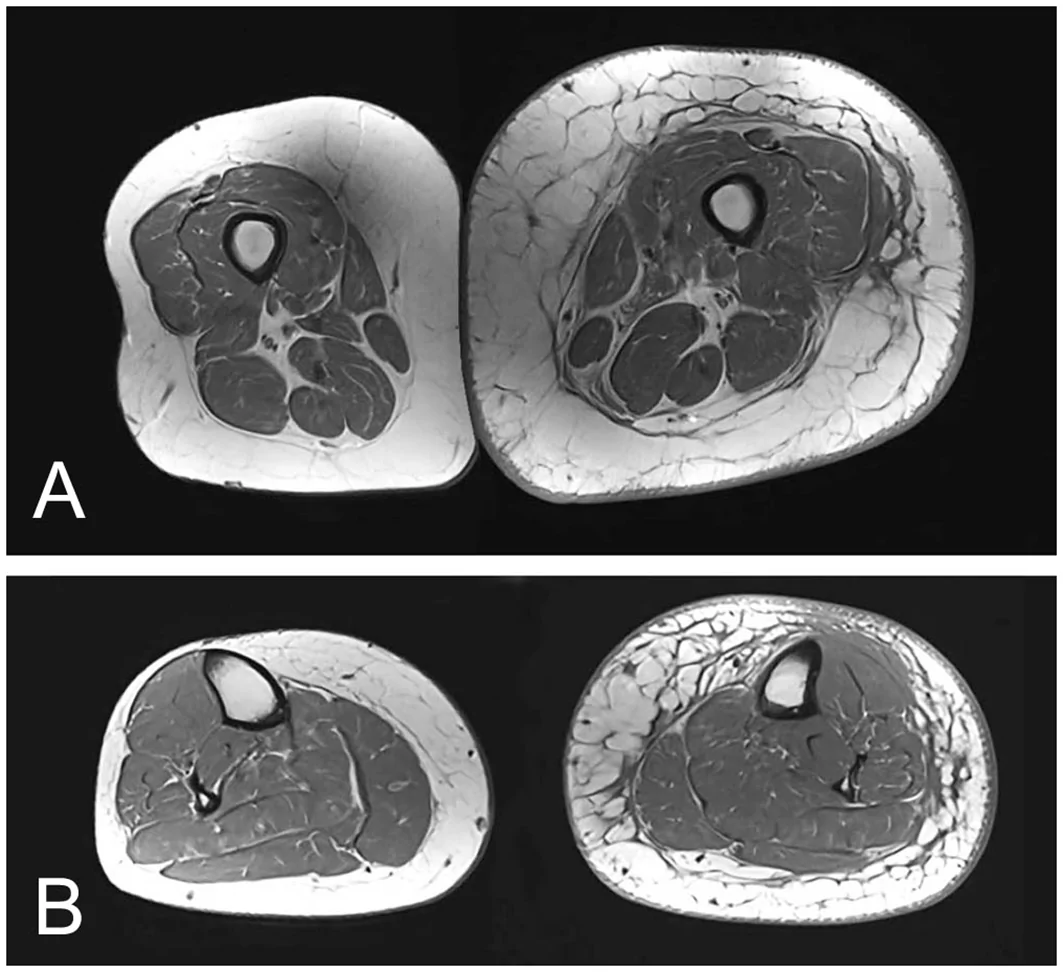
T1 MRI images of the lymphedema patient in **Figure 6**, 30 cm above the knee **(A)** and 30 cm below the knee **(B)**. The left lower limb volume is 222,451 mL and the right lower limb volume is 14,332 mL, a volume difference of 57%.

### 2.3 Prevalence between women and men

The majority of lymphedema patients are female (Grigorean et al., 2023). This is partly due to secondary lymphedema related to breast and gynecologic cancers. However, primary lymphedema also occurs predominantly in females, suggesting inherent sex‐related predisposition (Smeltzer et al., 1985). Endocrine mechanism differences between sexes are generally referred to as explaining the prevalence in females; sex hormones, especially estrogen, can influence lymphatic endothelial biology and lymphangiogenesis, and may contribute to the female predominance of lymphedema (Morfoisse et al., 2021).

In addition, women generally possess more subcutaneous adipose tissue in the limbs than men, whereas men tend to accumulate visceral rather than subcutaneous fat (Rodrigues et al., 2025) (**Figures 8**, **9**). Given their greater subcutaneous adipose tissue volume, women may generate more interstitial fluid under baseline conditions. This could increase lymphatic workload and lower the threshold for lymphedema onset, contributing to the higher prevalence of lymphedema in females. Although no quantitative study has demonstrated sex differences in the diameter of collecting lymphatic vessels, our cadaveric observations suggest that lymphatic vessels are larger in men. This may imply greater transport capacity in men than women, which would be a protective factor against the development of lymphedema.

**Figure 8:**
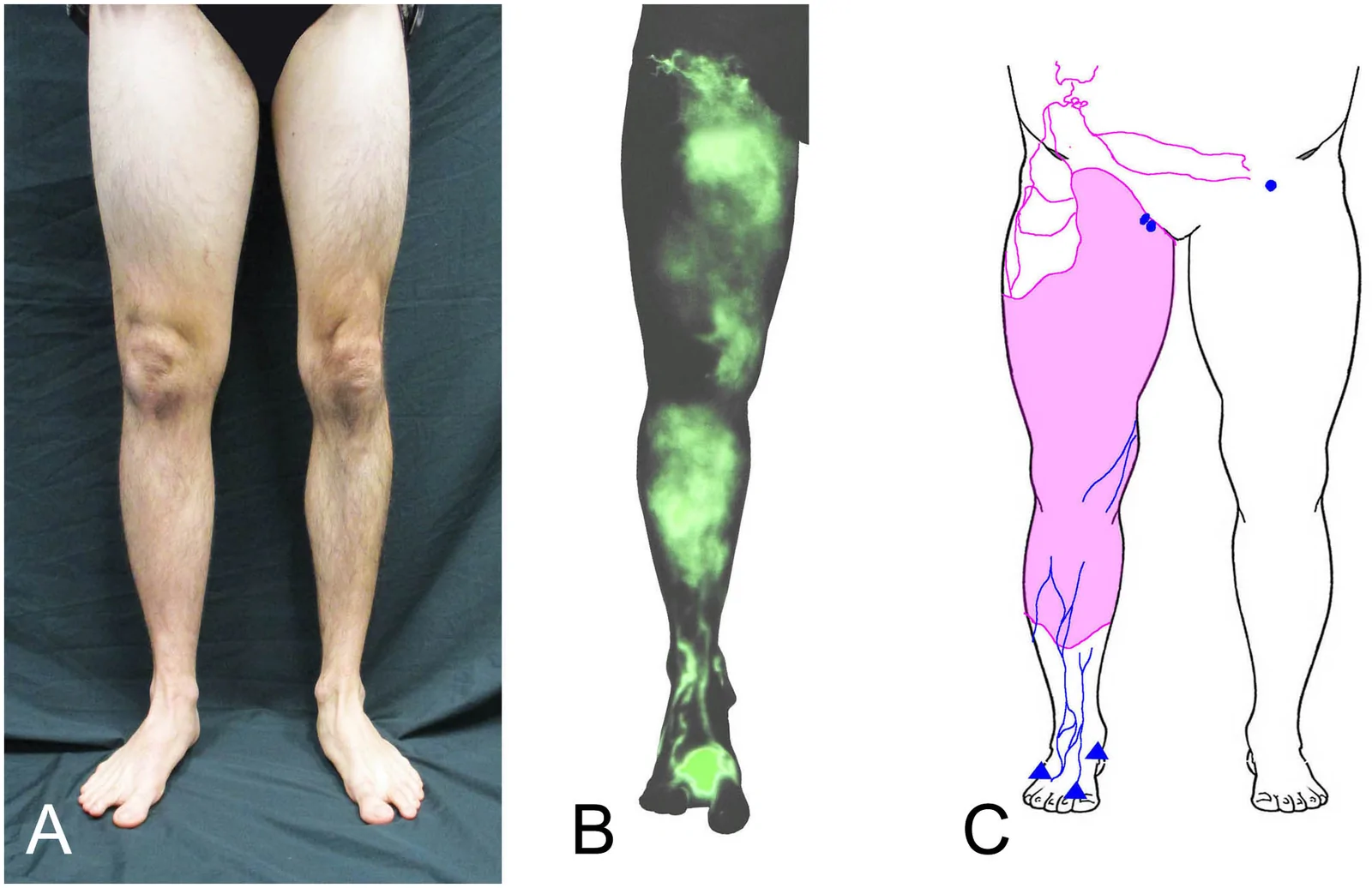
ICG lymphography images of a lean male patient (BMI 21) with unilateral secondary lymphedema in the right lower limb (MDACC Stage 3). From left: patient photo **(A)**, ICG montage image **(B)** and schematic diagram **(C)** (collecting lymph vessel: blue, dermal backflow: pink).

**Figure 9:**
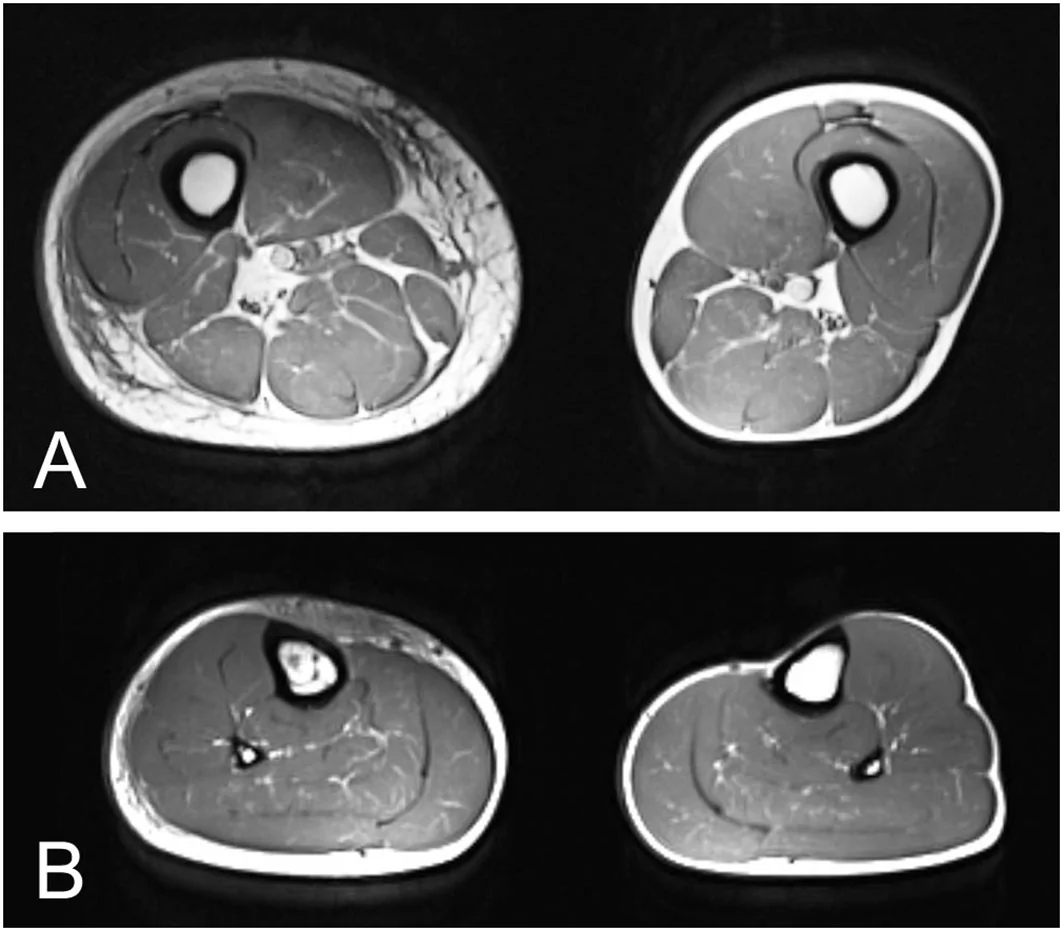
T1 MRI images of the lymphedema patient in **Figure 8**, 30 cm above the knee **(A)** and 30 cm below the knee **(B)**. The subcutaneous adipose tissue volume in the left lower limb of the male is thinner than in females.

### 2.4 Onset of primary lymphedema during puberty

The pathological mechanisms underlying onset of primary lymphedema during adolescence remain an unsolved question, though hormonal transition has been proposed. Kinmonth used a radiographic lymphangiography technique and identified diverse patterns of lymphatic abnormalities in primary lower limb lymphedema, including hypoplastic, hyperplastic and degenerative changes in the inguinal lymph nodes (Kinmonth, 1965). They identified that the hypoplastic type was the most frequent form of primary lymphedema. Similarly, our ICG lymphography examinations demonstrated fewer lymphatic vessels in the lower limbs in cases of adolescent‐onset primary lymphedema (**Figure 10**). Development of the lymphatic system is completed at birth, and thus one assumption is that congenital lymphatic hypoplasia remains subclinical until adolescence.

**Figure 10:**
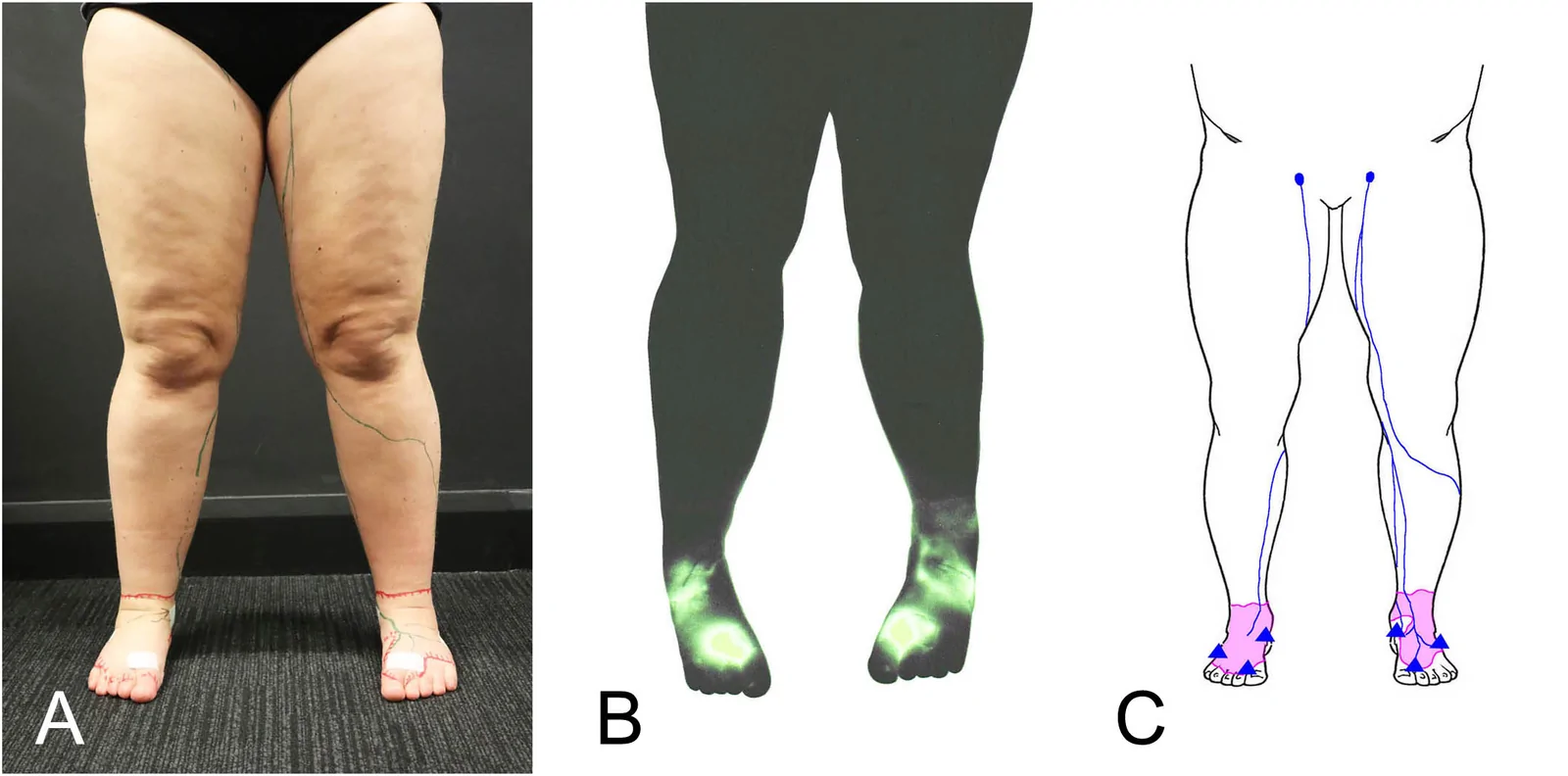
ICG lymphography images of a young female patient (BMI 33) with an 8-month history of bilateral primary lymphedema (MDACC Stage 1). From the left: patient photo **(A)**, ICG montage image **(B)** and schematic diagram **(C)** (collecting lymph vessel: blue, dermal backflow: pink). Note the lower number of lymphatic vessels in each limb compared to those in the right lower limb of the patient in **Figure 4**.

Children have undifferentiated adipose tissue and shorter limbs. During adolescence, adipocytes differentiate and increase in number, subcutaneous adipose volume increases, and the lower limbs lengthen rapidly. These anatomical changes increase lymphatic workload by increasing both interstitial fluid production and the transport distance against gravity. These factors increase the burden on lymphatic transport, so the number of lymphatic vessels that had sufficed in childhood may become insufficient in adolescence. The significant hormonal changes that occur in puberty can also impact the lymphatic system. The onset of primary lymphedema may therefore reflect an interaction between increasing adipose tissue burden, developmental limitations of the lymphatic network, and hormonal transition.

## 3 Discussion

The anatomy of the lymphatic system has been described as highly variable between individuals. However, recent updates with the microinjection technique in a cadaver model and ICG lymphography in clinical settings have refined our understanding further (Suami et al., 2005, 2019, 2022). The lymphosome concept was proposed to provide pictorial identities. Anatomical variations in lymphatic pathways are largely uniform in terms of focusing on the relationship between the collecting lymph vessels and their corresponding lymph nodes (Suami and Scaglioni, 2018).

The traditional physiological concept of lymphedema is an imbalance between interstitial fluid production and lymphatic drainage, and this remains valid (Mortimer and Rockson, 2014). Interstitial fluid formation is regulated by multiple physiological factors, including vascular filtration, permeability, hydrostatic and oncotic pressures, extracellular matrix properties, and lymphatic uptake (Wiig and Swartz, 2012). Lymphatic dysfunction initiates a feed-forward cycle in which impaired lymphatic transport capacity induces chronic inflammation and tissue fibrosis (Savetsky et al., 2014). These changes further remodel affected tissues and may contribute to heterogeneous presentation of lymphedema. However, to date, there have not been established biomarkers to measure these parameters in clinical settings. In this article, we explored unresolved aspects of lymphedema physiology by hypothesizing that the volume of subcutaneous adipose tissue can be an indicator of interstitial fluid production, and the number of lymphatic vessels can influence drainage capacity. Our observational findings supported the theory that baseline subcutaneous adipose volume contributes to the manifestation and onset of lymphedema, and may be considered a potential biomarker in clinical settings.

Canine models were used for lymphedema studies in the past, and rodent models are now prevalent. Both species have minimal subcutaneous adipose tissue, which may explain the difficulty in representing chronic human lymphedema. Species with greater limb adiposity, such as swine or ovine species, may serve more anatomically relevant models.

Limb circumference or truncated limb volume calculation is a standard method to assess and monitor lymphedema, due to its simplicity (Karges et al., 2003). However, this type of measurement includes entire tissues and therefore sensitivity decreases, because lymphedema primarily affects the skin and subcutaneous tissues above the deep fascia. More precise assessment can be achieved by comparing the volume of the subcutaneous adipose tissue between affected and unaffected limbs in cases of unilateral limb lymphedema. Our findings indicated that women with lower baseline subcutaneous adipose tissue volume manifest less adipose hypertrophy when lymphedema develops. Imaging subcutaneous adipose tissue in the unaffected limb with MRI may help predict lymphedema severity early in its course. Prospective collection of MRI images of the limbs during initial cancer treatment, and assessment of the subcutaneous tissue volume, may help predict the likelihood and prognosis of limb lymphedema. This hypothesis provides theoretical support for weight‐loss interventions by helping lymphedema patients better understand the rationale behind such therapies. This anatomy-based hypothesis provides a unifying explanation for several unsolved clinical observations, including the difficulty of reproducing chronic lymphedema in animal models, the protective effect of leanness, sex differences, and adult-onset primary lymphedema.

## Data Availability

The raw data supporting the conclusions of this article will be made available by the authors, without undue reservation.
